# 3,3′-Diphenyl-1,1′-(butane-1,4-di­yl)dithio­urea

**DOI:** 10.1107/S1600536811033071

**Published:** 2011-08-27

**Authors:** Pramod Pansuriya, Holger B. Friedrich, Glenn E. M. Maguire

**Affiliations:** a School of Chemistry, University of KwaZulu-Natal, Durban 4000, South Africa

## Abstract

The asymmetric unit of the title compound, C_18_H_22_N_4_S_2_, contains one half-mol­ecule, the complete mol­ecule being generated by crystallographic inversion symmetry. The crystal structure features two inter­molecular N—H⋯S hydrogen-bonding inter­actions, the first generating an infinite chain along the *b* axis and the second an infinite chain along the *a* axis, together forming an inter­locking structure.

## Related literature

Thio­urea derivatives are conspicuous for their biological activity as they form strong hydrogen-bonding inter­actions and coordinate to metal ions, see: Wittkopp & Schreiner (2003 ▶); Li *et al.* (2008 ▶). For appliactions of thio­urea, see Abdallah *et al.* (2006 ▶); Karamé *et al.* (2003 ▶); Nan *et al.* (2000 ▶); Breuzard *et al.* (2000 ▶); Tommasino *et al.*, (2000 ▶); Reinoso García *et al.* (2004 ▶); Leung *et al.* (2008 ▶). For synthesis of the title compound, see: Lee *et al.* (1985 ▶).
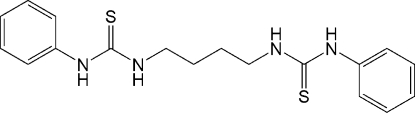


## Experimental

### 

#### Crystal data

C_18_H_22_N_4_S_2_
*M* *_r_* = 358.52
Monoclinic, 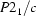

*a* = 9.6795 (3) Å
*b* = 7.8677 (3) Å
*c* = 12.3213 (4) Å
β = 105.816 (2)°
*V* = 902.81 (5) Å^3^
*Z* = 2
Mo *K*α radiation
μ = 0.30 mm^−1^
*T* = 173 K
0.46 × 0.45 × 0.13 mm

#### Data collection

Bruker APEXII CCD diffractometer
9210 measured reflections
2192 independent reflections
1710 reflections with *I* > 2σ(*I*)
*R*_int_ = 0.042

#### Refinement

*R*[*F* ^2^ > 2σ(*F* ^2^)] = 0.034
*wR*(*F* ^2^) = 0.093
*S* = 1.06
2192 reflections
117 parameters
H atoms treated by a mixture of independent and constrained refinement
Δρ_max_ = 0.45 e Å^−3^
Δρ_min_ = −0.23 e Å^−3^

### 

Data collection: *APEX2* (Bruker, 2006 ▶); cell refinement: *SAINT-Plus* (Bruker, 2006 ▶); data reduction: *SAINT-Plus*; program(s) used to solve structure: *SHELXS97* (Sheldrick, 2008 ▶); program(s) used to refine structure: *SHELXL97* (Sheldrick, 2008 ▶); molecular graphics: *OLEX2* (Dolomanov *et al.*, 2009 ▶); software used to prepare material for publication: *SHELXTL* (Sheldrick, 2008 ▶).

## Supplementary Material

Crystal structure: contains datablock(s) I, global. DOI: 10.1107/S1600536811033071/om2458sup1.cif

Structure factors: contains datablock(s) I. DOI: 10.1107/S1600536811033071/om2458Isup2.hkl

Supplementary material file. DOI: 10.1107/S1600536811033071/om2458Isup3.cml

Additional supplementary materials:  crystallographic information; 3D view; checkCIF report

## Figures and Tables

**Table 1:** Hydrogen-bond geometry (Å, °)

*D*—H⋯*A*	*D*—H	H⋯*A*	*D*⋯*A*	*D*—H⋯*A*
N1—H1*N*⋯S1^i^	0.855 (18)	2.508 (18)	3.3465 (13)	167.1 (15)
N2—H2*N*⋯S1^ii^	0.806 (15)	2.713 (16)	3.3755 (14)	140.7 (13)

Symmetry codes: (i) 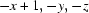; (ii) 
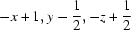
.

**Fig. 1.**
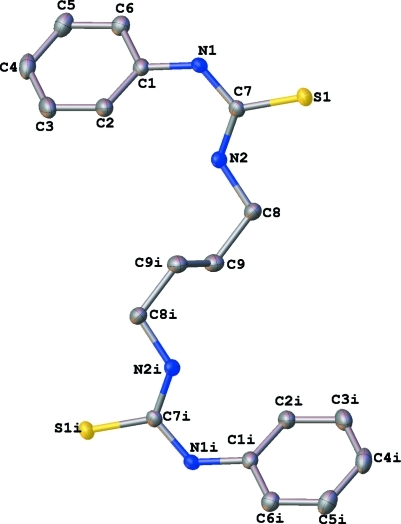
The molecular structure of the title compound with atomic numbering scheme. The H atoms have been omitted for clarity. Displacement ellipsoids are drawn at 40% probability. The 1,1'-(butane-1,4-diyl)bis(3-phenylthiourea) has inversion symmetry [symmetry code: (i): 1 - x, -y, 1-z].

**Fig. 2.**
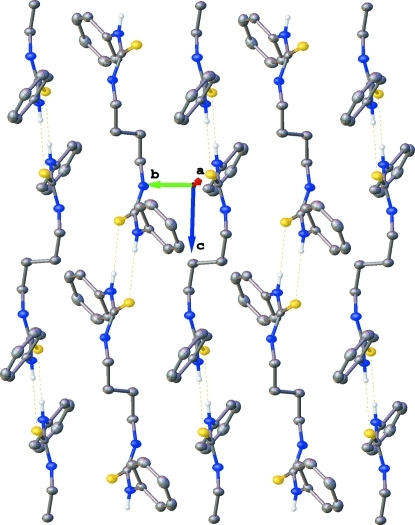
The hydrogen bonding interactions of the title compound as viewed down the a axis. All H atoms except those involved in hydrogen bonding interactions have been omitted for clarity.

## supplementary crystallographic information

### Comment

Thiourea derivatives are conspicuous for their biological activity as they form strong hydrogen bonding interactions and coordinate metal ions (Wittkopp & Schreiner, 2003; Li *et al.*, 2008). In recent years the use of thiourea groups as potential catalytic ligands has been extensively studied in reactions such as hydroformylation (Abdallah *et al.*, 2006), hydrosilylation (Karamé *et al.*, 2003), asymmetric reduction (Nan *et al.*, 2000), cyclization (Breuzard *et al.*, 2000) and hydrogenation (Tommasino *et al.*, 2000). Other applications include their use as synthetic cation-anion ionophores (Reinoso García *et al.*, 2004; Leung *et al.*, 2008).

Here we report the crystal structure of the title compound (Lee *et al.*, 1985) (Fig. 1). The structure shows two distinct intermolecular hydrogen bonding interactions. The first occurs between between N1–H1 and S1 2.508 (18) Å, that creates an infinite chain of molecules along the *b* axis. The second occurs between N2–H2 and S1 2.713 (16) Å, that generates an infinite chain along the *a* axis. Due to these interactions an interlocking molecular structure is formed (Fig. 2).

### Experimental

A solution of phenyl isothiocyanate (6.75 g, 50 mmol) in diethyl ether (15 ml) was added dropwise at 15°C to a vigorously stirred solution of anhydrous butane-1,4-diamine (8.81 g, 100 mmol) in isopropyl alcohol (100 ml) over a period of 30 min.The reaction mixture was stirred for 2 hrs at room temperature and quenched with water (200 ml). The reaction mixture was maintained overnight at room temperature. Then the reaction mixture was acidified with conc. HCl up to pH 2.6. The solvents were evaporated under vacuum, the residue was suspended in hot water for 30 min and the resulting precipitate was filtered off. The product was washed with ice cold water and dried. The yield was 2.36 g (35%).

Crystals suitable for single-crystal X-ray diffraction were grown in methanol: methylene chloride (1:2) at room temperature. *M*.p. = 458 K.

^1^H NMR (CDCl_3_, 400 MHz) d (p.p.m.): 7.64 (br.s., 2H, NH—CS), 7.40- 7.46 (m, 4H, H-arom), 7.29–7.33 (t, 2H, H-arom), 7.19–7.21 (d, 4H, H-arom), 6.18 (br.s., 2H, –NH—CH_2_), 3.65 (m, 4H, –CH_2_–CH_2_), 1.61 (m, 4H, –CH_2_–CH_2_).

^13^C NMR (CDCl_3_, 400 MHz): 26.12, 44.75, 125.45, 127.55, 130.34, 180.92

IR. (ν, cm^-1^) 3155, 3005, 2933, 1591, 1518, 1492, 1294, 1254, 1178,1071.

### Refinement

With the exception of those involved in hydrogen bonding, all hydrogen atoms were first located in the difference map then positioned geometrically and allowed to ride on their respective parent atoms with C—H = 0.95Å and *U_i_*_so_(H) = 1.2*U*_eq_(C) for aromatic and C—H = 0.99Å and *U_i_*_so_(H) = 1.2*U*_eq_(C) for CH_2_. Hydrogen atoms involved in hydrogen bonding were located in the difference map and refined freely.

### Crystal data

**Table:**

C_18_H_22_N_4_S_2_	*F*(000) = 380
*M**_r_* = 358.52	*D*_x_ = 1.319 Mg m^−^^3^
Monoclinic, *P*2_1_/*c*	Mo *K*α radiation, λ = 0.71073 Å
Hall symbol: -P 2ybc	Cell parameters from 3394 reflections
*a* = 9.6795 (3) Å	θ = 3.1–28.2°
*b* = 7.8677 (3) Å	µ = 0.30 mm^−^^1^
*c* = 12.3213 (4) Å	*T* = 173 K
β = 105.816 (2)°	Plate, colourless
*V* = 902.81 (5) Å^3^	0.46 × 0.45 × 0.13 mm
*Z* = 2	

### Data collection

**Table:**

Bruker APEXII CCD diffractometer	1710 reflections with *I* > 2σ(*I*)
Radiation source: fine-focus sealed tube	*R*_int_ = 0.042
graphite	θ_max_ = 28.0°, θ_min_ = 2.2°
φ and ω scans	*h* = −12→12
9210 measured reflections	*k* = −10→10
2192 independent reflections	*l* = −16→16

### Refinement

**Table:**

Refinement on *F*^2^	Primary atom site location: structure-invariant direct methods
Least-squares matrix: full	Secondary atom site location: difference Fourier map
*R*[*F*^2^ > 2σ(*F*^2^)] = 0.034	Hydrogen site location: inferred from neighbouring sites
*wR*(*F*^2^) = 0.093	H atoms treated by a mixture of independent and constrained refinement
*S* = 1.06	*w* = 1/[σ^2^(*F*_o_^2^) + (0.0513*P*)^2^ + 0.0075*P*] where *P* = (*F*_o_^2^ + 2*F*_c_^2^)/3
2192 reflections	(Δ/σ)_max_ = 0.005
117 parameters	Δρ_max_ = 0.45 e Å^−^^3^
0 restraints	Δρ_min_ = −0.23 e Å^−^^3^

### Special details

**Table:**

Geometry. All e.s.d.'s (except the e.s.d. in the dihedral angle between two l.s. planes) are estimated using the full covariance matrix. The cell e.s.d.'s are taken into account individually in the estimation of e.s.d.'s in distances, angles and torsion angles; correlations between e.s.d.'s in cell parameters are only used when they are defined by crystal symmetry. An approximate (isotropic) treatment of cell e.s.d.'s is used for estimating e.s.d.'s involving l.s. planes.
Refinement. Refinement of *F*^2^ against ALL reflections. The weighted *R*-factor *wR* and goodness of fit *S* are based on *F*^2^, conventional *R*-factors *R* are based on *F*, with *F* set to zero for negative *F*^2^. The threshold expression of *F*^2^ > σ(*F*^2^) is used only for calculating *R*-factors(gt) *etc*. and is not relevant to the choice of reflections for refinement. *R*-factors based on *F*^2^ are statistically about twice as large as those based on *F*, and *R*- factors based on ALL data will be even larger.

### Fractional atomic coordinates and isotropic or equivalent isotropic displacement parameters (Å^2^)

**Table:**

	*x*	*y*	*z*	*U*_iso_*/*U*_eq_	
C1	0.23226 (14)	−0.10682 (19)	0.10971 (12)	0.0250 (3)	
C2	0.17043 (15)	−0.0394 (2)	0.19000 (13)	0.0314 (4)	
H2	0.2223	0.0392	0.2445	0.038*	
C3	0.03242 (16)	−0.0880 (2)	0.18976 (14)	0.0383 (4)	
H3	−0.0092	−0.0449	0.2457	0.046*	
C4	−0.04488 (16)	−0.1990 (2)	0.10837 (15)	0.0393 (4)	
H4	−0.1387	−0.2333	0.1092	0.047*	
C5	0.01453 (16)	−0.2598 (2)	0.02594 (14)	0.0366 (4)	
H5	−0.0397	−0.3326	−0.0315	0.044*	
C6	0.15346 (15)	−0.2145 (2)	0.02723 (12)	0.0296 (3)	
H6	0.1947	−0.2578	−0.0289	0.036*	
C7	0.49385 (14)	−0.05136 (17)	0.19228 (11)	0.0223 (3)	
C8	0.60322 (15)	−0.0926 (2)	0.39648 (12)	0.0286 (3)	
H8A	0.6645	0.0072	0.3938	0.034*	
H8B	0.6631	−0.1961	0.4027	0.034*	
C9	0.54395 (17)	−0.0793 (2)	0.49881 (12)	0.0316 (3)	
H9A	0.4833	−0.1801	0.5004	0.038*	
H9B	0.6253	−0.0821	0.5680	0.038*	
N1	0.37258 (12)	−0.05795 (17)	0.10580 (10)	0.0265 (3)	
N2	0.48550 (13)	−0.10013 (17)	0.29351 (10)	0.0261 (3)	
S1	0.65024 (4)	0.01605 (5)	0.16882 (3)	0.02773 (13)	
H1N	0.3820 (18)	−0.045 (2)	0.0393 (15)	0.036 (5)*	
H2N	0.4173 (16)	−0.158 (2)	0.2950 (13)	0.028 (4)*	

### Atomic displacement parameters (Å^2^)

**Table:**

	*U*^11^	*U*^22^	*U*^33^	*U*^12^	*U*^13^	*U*^23^
C1	0.0203 (7)	0.0312 (8)	0.0236 (7)	0.0032 (6)	0.0058 (5)	0.0059 (6)
C2	0.0244 (7)	0.0448 (9)	0.0253 (8)	0.0052 (6)	0.0073 (6)	0.0008 (6)
C3	0.0274 (8)	0.0553 (11)	0.0354 (9)	0.0102 (7)	0.0140 (7)	0.0074 (8)
C4	0.0216 (7)	0.0464 (10)	0.0506 (10)	0.0014 (7)	0.0110 (7)	0.0127 (8)
C5	0.0254 (8)	0.0352 (9)	0.0450 (10)	−0.0020 (6)	0.0024 (7)	0.0003 (7)
C6	0.0260 (7)	0.0321 (8)	0.0301 (8)	0.0030 (6)	0.0062 (6)	0.0008 (6)
C7	0.0219 (7)	0.0238 (7)	0.0225 (7)	0.0013 (5)	0.0083 (5)	−0.0036 (5)
C8	0.0223 (7)	0.0392 (8)	0.0230 (7)	0.0017 (6)	0.0041 (6)	−0.0015 (6)
C9	0.0311 (8)	0.0392 (9)	0.0234 (7)	0.0028 (7)	0.0057 (6)	0.0023 (6)
N1	0.0221 (6)	0.0406 (7)	0.0181 (6)	−0.0017 (5)	0.0076 (5)	0.0001 (5)
N2	0.0204 (6)	0.0368 (7)	0.0215 (6)	−0.0068 (5)	0.0064 (5)	0.0008 (5)
S1	0.0215 (2)	0.0385 (2)	0.0256 (2)	−0.00221 (15)	0.01058 (15)	−0.00035 (15)

### Geometric parameters (Å, °)

**Table:**

C1—C6	1.382 (2)	C7—N2	1.3283 (17)
C1—C2	1.393 (2)	C7—N1	1.3543 (18)
C1—N1	1.4250 (17)	C7—S1	1.7014 (14)
C2—C3	1.389 (2)	C8—N2	1.4572 (18)
C2—H2	0.9500	C8—C9	1.5246 (19)
C3—C4	1.385 (2)	C8—H8A	0.9900
C3—H3	0.9500	C8—H8B	0.9900
C4—C5	1.382 (2)	C9—C9^i^	1.516 (3)
C4—H4	0.9500	C9—H9A	0.9900
C5—C6	1.387 (2)	C9—H9B	0.9900
C5—H5	0.9500	N1—H1N	0.855 (18)
C6—H6	0.9500	N2—H2N	0.806 (15)
			
C6—C1—C2	119.85 (13)	N1—C7—S1	119.92 (10)
C6—C1—N1	118.73 (12)	N2—C8—C9	109.98 (11)
C2—C1—N1	121.28 (13)	N2—C8—H8A	109.7
C3—C2—C1	119.53 (15)	C9—C8—H8A	109.7
C3—C2—H2	120.2	N2—C8—H8B	109.7
C1—C2—H2	120.2	C9—C8—H8B	109.7
C4—C3—C2	120.30 (15)	H8A—C8—H8B	108.2
C4—C3—H3	119.9	C9^i^—C9—C8	114.35 (16)
C2—C3—H3	119.9	C9^i^—C9—H9A	108.7
C5—C4—C3	119.97 (14)	C8—C9—H9A	108.7
C5—C4—H4	120.0	C9^i^—C9—H9B	108.7
C3—C4—H4	120.0	C8—C9—H9B	108.7
C4—C5—C6	119.93 (15)	H9A—C9—H9B	107.6
C4—C5—H5	120.0	C7—N1—C1	127.84 (12)
C6—C5—H5	120.0	C7—N1—H1N	117.0 (12)
C1—C6—C5	120.33 (14)	C1—N1—H1N	114.6 (12)
C1—C6—H6	119.8	C7—N2—C8	124.94 (12)
C5—C6—H6	119.8	C7—N2—H2N	116.4 (11)
N2—C7—N1	117.79 (12)	C8—N2—H2N	117.0 (11)
N2—C7—S1	122.29 (11)		
			
C6—C1—C2—C3	3.2 (2)	N2—C8—C9—C9^i^	−62.1 (2)
N1—C1—C2—C3	178.86 (14)	N2—C7—N1—C1	2.1 (2)
C1—C2—C3—C4	−1.8 (2)	S1—C7—N1—C1	−178.44 (12)
C2—C3—C4—C5	−1.0 (3)	C6—C1—N1—C7	−135.44 (15)
C3—C4—C5—C6	2.3 (3)	C2—C1—N1—C7	48.9 (2)
C2—C1—C6—C5	−1.9 (2)	N1—C7—N2—C8	−176.58 (13)
N1—C1—C6—C5	−177.63 (14)	S1—C7—N2—C8	4.0 (2)
C4—C5—C6—C1	−0.9 (2)	C9—C8—N2—C7	154.26 (15)

Symmetry codes: (i) −*x*+1, −*y*, −*z*+1.

### Hydrogen-bond geometry (Å, °)

**Table:**

*D*—H···*A*	*D*—H	H···*A*	*D*···*A*	*D*—H···*A*
N1—H1N···S1^ii^	0.855 (18)	2.508 (18)	3.3465 (13)	167.1 (15)
N2—H2N···S1^iii^	0.806 (15)	2.713 (16)	3.3755 (14)	140.7 (13)

Symmetry codes: (ii) −*x*+1, −*y*, −*z*; (iii) −*x*+1, *y*−1/2, −*z*+1/2.
